# Evolution in the Bottling of Cabernet Sauvignon Wines Macerated with Their Own Toasted Vine-Shoots

**DOI:** 10.1021/acs.jafc.2c08978

**Published:** 2023-03-29

**Authors:** C. Cebrián-Tarancón, R. Sánchez-Gómez, F. Fernández-Roldán, G. L. Alonso, M. R. Salinas

**Affiliations:** † Cátedra de Química Agrícola, E.T.S.I. Agrónomos y Montes, Universidad de Castilla-La Mancha, Avda. de España s/n, 02071 Albacete, Spain; ‡ Pago de la Jaraba, Crta, Nacional 310, km 142, 7, 02600 Villarrobledo, Spain

**Keywords:** bottle aging, enological additive, volatile, phenolic, sensory profile, SEGs

## Abstract

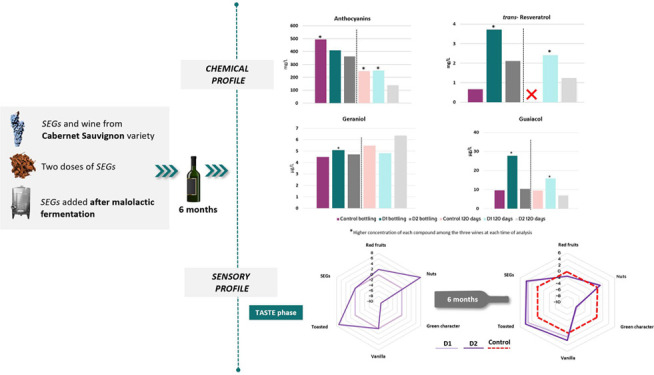

This work studies, for the first time, the effect of the use of Cabernet Sauvignon vine-shoots as an enological additive (called “Shoot Enological Granule”, SEG) in wines of the same variety. SEGs were added in two doses (12 and 24 g/L) at the end of malolactic fermentation, and after that, wines were bottled for six months. The phenolic and volatile composition and sensory profiles of wines were analyzed at bottling and after six months. The results showed a decrease in the total content of phenolic compounds with bottle time; however, stilbenes—specifically *trans*-resveratrol—were maintained at significant levels in SEG wines. In contrast, the total content of volatile compounds, mainly esters, increased with bottle aging. Finally, in terms of sensory profile, SEG wines showed a clear differentiation between the descriptors and the control, with more-integrated aromas after bottle time with more toasted, nutty vanilla notes, as well as silkier and less bitter tannins, compared to the control.

## 1 Introduction

Nowadays, the wine sector is subject to constant change. The search for new enological tools to achieve differentiated wines has aroused great interest among winemakers. As such, the use of toasted vine-shoots in winemaking has been extensively studied in recent years. Toasted vine-shoots have been proposed as enological additives due to them being a simple and economical tool for developing wines of high chemical and organoleptic quality.^1−3^

With regard to the use of toasted vine-shoots in winemaking, their effect on the chemical composition of wine has already been tested in white varieties such as Airén^2^ and red varieties such as Cencibel,^2^ Malbec, or Bonarda.^4^ These works have studied the impact of various factors (fragment size, dosage, contact time, or addition moment) on the chemical profile of wines, concluding that the effect is not dependent on a single factor but on the interaction of dose and moments of addition during winemaking. However, other varieties widely used in viticulture have not been studied until now, such as Cabernet Sauvignon, whose vine-shoots could have a good enological aptitude. Even pruned, toasted vine-shoots have been assigned a specific name, such as SEG, which is a term derived from the “Shoot from vines – Enological – Granule”.^1^ The evolution of vineyard pesticides during the transformation from vine-shoots to SEGs (mainly during storage and toasting) was studied, being, in all cases, below their respective maximum residue limits (MRLs),^5^ as well as their possible toxicity and cytotoxicity.^6^ Moreover, a classification method based on their enological aptitude has been proposed.^7^

It is well-known that bottle aging is an important part of the wine production process that affects the evolution of color, aroma, mouthfeel, and taste of wine, since, during the bottle aging, the chemical composition of wines is modified due to numerous chemical reactions that modify the wine quality. Thus, for example, anthocyanins interact with flavanols, flavonols, or tannins and are progressively transformed to more stable polymeric pigments, which give rise to changes in the color or astringency and bitterness of wines.^8−10^ Moreover, in terms of sensory and aroma profile, it has been demonstrated that, during the wine bottle aging process, an evolution of primary and secondary aromas increases the complexity of the wine by potentially forming positive aroma compounds or releasing compounds from their glycoside precursors.^11−14^ These aforementioned phenomena alter the composition of the wine, thus changing its sensory attributes.^15^ However, how these aspects relate to the evolution of bottles of wine in contact with vine-shoots is still unknown. Therefore, based on the above, the aim of this work is to study the evolution of the chemical and sensory profile of Cabernet Sauvignon wines elaborated in contact with SEGs of the same variety added in different doses after malolactic fermentation.

## 2 Materials and Methods

### 2.1 Plant Material

Vine-shoots were pruned in January 2020 from Cabernet Sauvignon red *Vitis vinifera L*. cultivar (CS; VIVC: 1929) grown in the Pago de La Jaraba winery (Castilla-La Mancha, Spain). The grapevines were planted as a vertical shoot position trellis, pruned to bilateral cordon, and were grown in an ecological system under nonirrigation conditions. After pruning, the samples were stored intact in darkness at room temperature (18 ± 3 °C) for six months and then ground into granules ranging from 2 mm to 2 cm, using a hammer miller (Skid Sinte 1000; LARUS Impianti, Zamora, Spain). Then, the samples were subjected to a toasting process in an oven with air circulation (Heraeus T6; Heraeus, Hanau, Germany) at 180 °C for 45 min, according to the method proposed by Cebrián-Tarancón et al.^16^

### 2.2 Winemaking with SEGs

Grapes were harvested at the optimum maturation moment from the same vineyards where the vine-shoots were pruned. Grape enological parameters were analyzed according to OIV methods^17^ and are summarized in Table S1 in the Supporting Information. Musts were inoculated simultaneously with the commercial *Saccharomyces cerevisiae* strain Uvaferm HPS active dry yeast (Lallemand, St. Simon, France) to perform the alcoholic fermentation (AF) and the commercial *Oenococcus oeni* strain Lalvin VP41 (Lallemand, St. Simon, France) to develop the malolactic fermentation (MF). Vinifications were performed in duplicate at a controlled temperature (20 ± 2 °C), using 500-L stainless steel tanks. Finally, potassium metabisulfite was added after malolactic fermentation to give a total SO_2_ concentration of 50 mg/L. Alcoholic fermentation, which lasted for 10 days, was followed by a daily determination of the must temperature and density. Malic acid was measured every 2 days, and when its concentration was below 0.1 g/L, malolactic fermentation was considered complete. SEGs were added to wines at a dosage of 12 and 24 g/L after malolactic fermentation and were removed after 30 days of maceration, according to Cebrián-Tarancón et al.^1^ Once malolactic fermentation had finished, wines were bottled and stored at 10 °C until their analysis. The mean values (± the standard deviation) of the enological parameters of wine at bottling time and after six months, analyzed according to OIV methods, are summarized in Table S2 in the Supporting Information.

The resulting wines were identified as follows: control analyzed at bottling (**C-B**), wine with 12 g/L of SEGs analyzed at bottling (**D1-B**), wine with 24 g/L of SEGs analyzed at bottling (**D2-B**), control analyzed six months after bottling (**C-6m**), wine with 12 g/L of SEGs analyzed six months after bottling (**D1-6m**), and wine with 24 g/L of SEGs analyzed six months after bottling (**D2-6m**).

### 2.4 Analysis

#### 2.4.1 Volatile Compounds Determined by SBSE-GC-MS

Wine volatiles were determined according to the methodology proposed by Sánchez-Gómez et al.^18^ Their extraction was conducted by means of stir bar sorptive extraction (SBSE) (PDMS; 10 mm length, 0.5 mm film thickness) after 25 mL of wine was stirred at 500 rpm for 60 min. Later, analysis was performed using an automated thermal desorption unit (TDU, Gerstel, Mülheim and der Ruhr, Germany) mounted on an Agilent 7890A gas chromatograph system (GC) coupled with a quadrupole Agilent 5975C electron ionization mass spectrometric detector (MS, Agilent Technologies, Palo Alto, CA, USA) equipped with a fused silica capillary column (BP21 stationary phase; 30 m length; 0.25 mm inner diameter (ID); and 0.25 μm film thickness) (SGE, Ringwood, Australia). The carrier gas was helium with a constant column pressure of 20.75 psi.

The stir bars were thermally desorbed in a stream of helium carrier gas at a flow rate of 75 mL/min with the TDU programmed from 40 °C to 295 °C (held for 5 min) at a rate of 60 °C/min in splitless desorption mode. The analytes were focused on a programmed temperature vaporizing injector (PTV) (CIS-4, Gerstel) containing a packed liner (20 mg tenax TA), held at −10 °C with cryo cooling prior to injection. After desorption and focusing, the CIS-4 was programmed from −10 °C to 260 °C (held for 5 min) at 12 °C/min to transfer the trapped volatiles onto the analytical column. The GC oven temperature was programmed to 40 °C (held for 2 min), raised to 80 °C (5 °C/min, held for 2 min), raised to 130 °C (10 °C/min, held for 5 min), raised to 150 °C (5 °C/min, held for 5 min), and then raised to 230 °C (10 °C/min, held for 5 min). The MS was operated in scan acquisition (27–300 *m*/*z*) with an ionization energy of 70 eV. The temperature of the MS transfer line was maintained at 230 °C. MS data acquisition was conducted in positive scan mode, although, to avoid matrix interferences, the MS quantification was performed in the single ion-monitoring mode using the characteristic *m*/*z* values of the compounds. Information related to analyzed compounds and *m*/*z* values are included in greater detail in Sánchez-Gómez et al.^18^ Compound identification was performed using the NIST library and confirmed by comparison with the mass spectra and retention time of their pure standards (Sigma–Aldrich, Steinheim, Germany). 3-Methyl-1-pentanol was used as an internal standard. Quantification was based on calibration curves of the respective standards at five different concentrations (*R*^2^ = 0.95–0.97). The analyses of each replicate of wine were made in triplicate.

#### 2.4.2 Low-Molecular-Weight Phenolic Compounds (LMWPCs) Determined by HPLC-DAD

The content of low-molecular-weight phenolic compounds (LMWPCs) of wines was determined according to Cebrián-Tarancón et al.^3^ A volume of 20 μL of wine was injected into an Agilent 1200 HPLC chromatograph (Palo Alto, CA, USA) equipped with a Diode Array Detector (DAD, Agilent G1315D) coupled to an Agilent ChemStation (version B.03.01) data-processing station. Separation was performed in a reverse phase ACE C18-PFP (4.6 mm × 150 mm, 3 μm particle size) and a precolumn ACE Excel HPLC Precolumn Filter 1PK (0.5 μm particle size) at 30 °C. The HPLC proportion of solvents used was water/formic acid/acetonitrile (97.5:1.5:1 v/v/v) as solvent A and acetonitrile/formic acid/solvent A (78.5:1.5:20 v/v/v) as solvent B. The elution gradient was set up for solvent B as 0 min, 5%; 8.40 min, 5%; 12.50 min, 10%; 19 min, 15%; 29 min, 16%; 30 min, 16.5%; 34.80 min, 18%; 37.20 min, 32%; 42 min, 62%; 52 min, 90%; 54 min, 100%; 56 min, 100%; 60 min, 5%; and 65 min, 5%. All compound detection was performed by means of a DAD detector via comparison with the corresponding ultraviolet-visible light (UV–Vis) spectra and retention time of the compounds’ pure standards (Sigma–Aldrich, Steinheim, Germany). The wavelength quantification of the analyzed compounds is outlined in greater detail in Cebrián-Tarancón et al.^3^ Quantification was based on the calibration curves of the respective standards at five different concentrations achieved by UV–Vis signal (*R*^2^ = 0.92–0.99). The analyses of each replicate of wine were made in duplicate.

#### 2.4.4 Sensory Analysis of the Wines

A group of nine expert panelists—three females and six males aged 25 to 65 years old—participated in the tasting. The training of the new descriptor SEGs consisted of offering the panelists food model wine (pH 3.6, 12.5% v/v alcoholic degree) with five doses of toasted SEGs, which were used as references to establish their intensity on a five-point scale (1 = not intense; 5 = very intense). Two sensorial analyses (at bottling and after six months) were carried out, evaluating in each of them the two wines from the treatments (bottling: **D1-B** and **D2-B**; 6 months **D1–6m** and **D2–6m**) and comparing them with respect to the control (**C–B** and **C-6m**). In each tasting, samples were presented in increasing order of SEGs quantity (**D1** and **D2**), and some information was given to the judges about the origin of the samples. All tastings were performed in an air-conditioned (21 °C) wine tasting room located in the cellar of at Pago de la Jaraba (Villarrobledo, Spain) at a round table with optimal conditions for facilitating the tasters’ sensory evaluation of the wine.

Since the aim was to evaluate the treatment effect, two bottles from the same treatments (one per repetition) were mixed prior to each tasting session. Thus, in each tasting moment, three wines were analyzed sensorially. Considering that SEGs can produce wine aromas that have not been defined until now, an adapted tasting evaluation sheet was created, including new descriptors. The odor attributes to evaluate were determined by consensus after the panel had discussed reducing the number of descriptors during a dedicated preliminary session. At each sampling time, each wine was assessed by judges in terms of 12 descriptors grouped by *visual phase* (purple, garnet, and red), *olfactory* and *taste phase* (red fruits, nuts, green character, vanilla, toasted, and *SEGs*) and *tannins* (dryness, silkiness, and bitterness).

Prior to the individual evaluation of each treatment, the evaluation of the control wine was performed jointly to establish a consensus assessment among all the tasters. Next, the panelists smelled and tasted the different wines, noted the specific perceived descriptors, and rated the intensity of each sensory descriptor on an 11-point scale, where 0 indicated that the descriptor was not perceived (absence) and values from 1 to 10 rated its intensity from very low to maximum, respectively.

### 2.5 Statistical Analysis

The statistical analysis of the results regarding wine composition and sensorial profile were examined using one-way analysis of variance (ANOVA) at a 95% probability level, according to the Fisher posthoc test, to determine the differences between the wines. These analyses were conducted using the Statgraphics Centurion statistical program (version 18.1.12; StatPoint, Inc., The Plains, VA, USA). A heatmap with dendrogram and principal component analysis (PCA) was also performed with the purpose of obtaining an overall view of the influence of the addition of SEGs and bottling aging on the chemical composition of wines. These data were processed using XLSTAT 2022 statistical software (Addinsoft, Paris, France).

## 3 Results and Discussion

### 3.1 Effect of SEGs in the Chemical Profile of Wines

The volatile and phenolic compositions of wines at both times of study (immediately before bottling and after six months of bottle aging) are summarized in Tables 1 and 2. The main volatile chemical families were esters, alcohols, and acids, and, in the case of phenolic compounds, anthocyanins and flavanols. The statistical information in the tables corresponds to two one-way ANOVA tests. First, for each treated wine, significant differences were found with respect to the control wine in each wine column. Second, the last two columns indicate the significant differences between the same dose (D1 or D2) but at a different time of analysis (bottling and six months after bottling).

**Table 1:** Volatile Compounds (μg/L) of Wines^a^

	C-B	D1-B	D2-B	C-6m	D1-6m	D2-6m	C-B/D1-B	C-B/D2-B	C-6m/D1-6m	C-6m/D2-6m	C-B/C-6m	D1-B/D1-6m	D2-B/D2-6m
**Acids**													
hexanoic acid	693.51 ± 100.54	946.93 ± 23.92	819.05 ± 348.48	1235.68 ± 382.38	1561.47 ± 294.45	2720.08 ± 143.29	11.58^b^	0.36	0.49	39.64^c^	5.64	4.17	76.37^d^
octanoic Acid	610.96 ± 49.13	605.97 ± 59.37	599.68 ± 70.80	1035.94 ± 260.41	981.33 ± 137.98	1160.42 ± 70.69	0.05	0.05	0.20	0.64	7.72^b^	6.00	94.24^d^
decanoic acid	80.65 ± 7.87	40.74 ± 4.45	55.51 ± 4.24	143.36 ± 36.65	67.11 ± 8.62	108.27 ± 9.59	34.98^c^	23.73^c^	7.98	2.57	8.40^b^	7.23	76.01^c^
**total Acids**	**1385.12 ± 155.46**	**1593.45 ± 87.74**	**1474.23 ± 418.84**	**2414.99 ± 675.78**	**2609.90 ± 441.05**	**3988.78 ± 222.47**	3.28	0.12	0.01	14.68	6.62	4.92	84.33^d^
**Alcohols**													
2-phenylethyl alcohol	4698.98 ± 115.26	7756.41 ± 991.36	4440.61 ± 318.24	6831.25 ± 1825.18	10041.54 ± 1278.63	8428.56 ± 596.09	26.82^b^	1.75	3.19	2.08	4.08	1.35	104.49^d^
1-hexanol	403.77 ± 7.78	610.68 ± 81.52	593.25 ± 9.81	619.68 ± 170.45	836.51 ± 147.46	1233.99 ± 47.83	18.95^b^	687.11^d^	1.21	36.12^c^	4.80	1.20	516.62^d^
benzyl alcohol	102.54 ± 1.72	174.16 ± 14.33	115.88 ± 5.20	148.58 ± 34.22	245.77 ± 28.99	258.66 ± 11.52	65.77^c^	17.83^b^	7.88	27.89^c^	5.42	4.45	382.68
nonanol	0.69 ± 0.06	1.91 ± 0.20	1.45 ± 0.09	1.06 ± 0.31	2.53 ± 0.39	2.78 ± 0.53	84.80^c^	140.75^d^	15.91^b^	23.96^c^	4.10	1.44	18.70^b^
**total Alcohols**	**5205.98 ± 122.08**	**8543.15 ± 1087.42**	**5151.19 ± 326.19**	**7600.57 ± 2023.54**	**11126.35 ± 1455.46**	**9924.00 ± 655.53**	14.98^b^	18.71^b^	2.48	7.23	1.54	0.04	93.91^d^
**Aldehydes**													
benzaldehyde	1.86 ± 0.17	3.92 ± 0.21	2.43 ± 0.21	3.56 ± 1.10	4.44 ± 1.25	3.56 ± 0.34	112.20^c^	13.50^b^	0.20	0.00	7.00	0.04	24.66^c^
nonanal	1.11 ± 0.01	1.27 ± 0.01	1.17 ± 0.05	2.29 ± 0.40	1.79 ± 0.01	1.77 ± 0.14	158.78^c^	4.73	2.88	4.48	25.97^c^	1193.74^d^	48.55^c^
2- phenyl acetaldehyde	0.97 ± 0.01	0.98 ± 0.03	0.93 ± 0.02	1.06 ± 0.10	1.27 ± 0.16	1.04 ± 0.03	1.02	12.88^b^	1.66	0.11	2.37	2.70	34.48^c^
**total aldehydes**	**3.94 ± 0.19**	**6.17 ± 0.18**	**4.54 ± 0.15**	**6.91 ± 1.59**	**7.49 ± 1.43**	**6.38 ± 0.46**	147.37^c^	18.71^b^	0.01	0.32	10.36^b^	0.59	43.43^c^
**Esters**													
***Ethyl Esters***													
ethyl lactate	33716.46 ± 1884.86	28537.47 ± 355.03	26421.53 ± 1029.53	94541.61 ± 13540.33	90329.11 ± 9540.19	137990.53 ± 9879.91	13.77^b^	34.61^c^	0.35	20.16^b^	59.39^c^	51.17^b^	378.45^d^
ethyl octanoate	16225.14 ± 528.55	25112.66 ± 3536.13	17597.60 ± 1665.94	22721.82 ± 5920.54	26366.86 ± 1883.06	31070.35 ± 3301.34	37.90^c^	1.89	0.55	3.15	3.58	0.01	39.82^c^
ethyl butyrate	190.93 ± 13.06	191.37 ± 10.17	177.68 ± 15.28	304.16 ± 35.53	324.94 ± 29.24	395.93 ± 9.73	0.08	1.31	0.14	18.62^b^	26.84^c^	20.78^b^	435.48^d^
ethyl decanoate	16.65 ± 0.62	13.48 ± 1.35	13.55 ± 1.09	23.48 ± 5.46	11.23 ± 0.60	22.48 ± 2.69	7.94^b^	18.40^b^	9.22	0.08	4.62	4.84	28.38^c^
diethyl succinate	43.29 ± 2.37	71.04 ± 6.60	41.76 ± 3.34	583.12 ± 154.04	927.94 ± 140.03	2403.73 ± 178.77	41.33^c^	0.42	4.34	178.56^d^	36.83^c^	45.02^b^	523.51^d^
ethyl vanillate	16.31 ± 1.96	36.65 ± 1.73	21.75 ± 1.03	66.55 ± 18.49	121.26 ± 20.21	81.82 ± 9.16	128.54^c^	18.18^b^	6.63	1.64	21.90^c^	19.85^b^	127.48^d^
ethyl hexanoate	20.06 ± 0.14	34.57 ± 6.27	25.38 ± 1.97	25.70 ± 6.70	38.32 ± 5.21	45.83 ± 5.52	16.27^b^	21.83^c^	3.41	16.14^b^	2.13	0.00	36.60^c^
ethyl cinnamate	0.07 ± 0.00	0.11 ± 0.02	0.07 ± 0.01	0.19 ± 0.06	0.22 ± 0.03	0.19 ± 0.02	16.30^b^	2.76	0.38	0.00	11.43^b^	12.52	98.33^d^
***Acetates***													
ethyl acetate	12740.67 ± 1555.80	14081.16 ± 641.35	8210.11 ± 2681.34	23185.09 ± 2869.24	25705.64 ± 3352.87	31618.32 ± 1142.68	1.54	6.41	0.26	22.37^c^	30.72^c^	12.55	193.50^d^
isoamyl acetate	461.82 ± 15.20	308.14 ± 8.88	362.26 ± 15.98	623.62 ± 55.36	448.57 ± 31.30	538.41 ± 22.54	141.68^c^	61.16^c^	16.06^b^	6.10	23.83^c^	20.91^b^	121.97^d^
2-phenylethyl acetate	18.52 ± 0.54	19.62 ± 3.07	14.35 ± 1.34	30.22 ± 7.89	32.59 ± 4.31	21.54 ± 1.92	0.97	25.01^c^	0.03	3.43	6.58	5.60	28.33^c^
hexyl acetate	4.51 ± 0.13	2.28 ± 0.02	4.47 ± 0.33	5.26 ± 0.53	2.46 ± 0.20	3.86 ± 0.07	481.84^d^	0.04	46.44^c^	20.06^b^	5.49	0.44	9.87^b^
**total esters**	**47290.96 ± 2190.95**	**43336.21 ± 319.18**	**35350.06 ± 3141.38**	**119435.26 ± 16454.18**	**117980.35 ± 13121.34**	**173155.07 ± 10847.81**	5.51	29.16^c^	0.12	22.29^c^	56.67^c^	38.76^b^	446.68^d^
**Norisoprenoids**													
β-ionol	22.05 ± 0.21	20.72 ± 0.20	20.62 ± 0.42	24.89 ± 1.36	22.24 ± 0.32	24.50 ± 0.42	39.42^c^	27.97^c^	7.06	0.19	12.87^b^	16.93	65.54^c^
β-damascenone	6.14 ± 0.02	6.36 ± 0.16	6.01 ± 0.06	8.13 ± 0.74	8.84 ± 0.42	9.00 ± 0.48	6.82	14.04^b^	0.92	2.95	21.39^c^	35.14^b^	115.39^d^
α-ionone	0.22 ± 0.01	0.22 ± 0.00	0.23 ± 0.01	0.23 ± 0.01	0.23 ± 0.02	0.24 ± 0.00	0.77	2.80	0.79	4.23	4.64	2.83	7.06
β-ionone	0.24 ± 0.00	0.29 ± 0.01	0.27 ± 0.00	0.25 ± 0.05	0.32 ± 0.01	0.33 ± 0.01	114.97^c^	161.12^c^	84.60^c^	101.41^d^	13.42^b^	5.54	61.05^c^
**total norisoprenoids**	**28.65 ± 0.22**	**27.60 ± 0.37**	**27.13 ± 0.45**	**33.50 ± 2.11**	**31.62 ± 0.76**	**34.07 ± 1.20**	10.59^b^	27.62^c^	1.62	0.16	15.68^b^	25.82^b^	87.69^d^
**Esters: Terpenes**													
geraniol	4.49 ± 0.17	5.08 ± 0.17	4.72 ± 0.13	5.47 ± 0.56	4.82 ± 1.01	6.36 ± 1.59	15.09^b^	3.79	0.21	0.84	8.44^b^	0.00	3.17
citronellol	2.07 ± 0.05	2.31 ± 0.22	1.92 ± 0.11	3.49 ± 0.70	3.55 ± 0.50	2.59 ± 0.06	4.31	4.85	0.02	4.97	12.31^b^	4.71	88.80^d^
farnesol	2.10 ± 0.09	1.95 ± 0.03	1.93 ± 0.06	2.82 ± 0.39	1.95 ± 0.07	2.16 ± 0.10	3.81	7.40^b^	8.86	7.74^b^	9.47^b^	0.22	12.89^b^
linalool	2.15 ± 0.01	2.30 ± 0.07	2.26 ± 0.01	2.72 ± 0.25	2.91 ± 0.19	2.94 ± 0.10	14.19^b^	122.50^c^	0.34	1.95	15.56^b^	9.28	137.73^d^
nerolidol	1.12 ± 0.01	1.17 ± 0.02	1.18 ± 0.03	1.30 ± 0.06	1.22 ± 0.02	1.32 ± 0.05	17.17^b^	11.48^b^	4.15	0.08	28.87^c^	2.10	16.29^b^
**total terpenes**	**11.92 ± 0.25**	**12.80 ± 0.50**	**12.01 ± 0.30**	**15.80 ± 1.75**	**14.44 ± 0.23**	**15.37 ± 1.68**	7.53	0.16	0.97	0.09	14.39^b^	10.74	11.58^b^
**Esters: Volatile Phenols**													
guaiacol	9.62 ± 1.56	27.69 ± 2.96	10.46 ± 0.47	9.45 ± 3.39	15.76 ± 3.73	6.97 ± 1.93	59.44^c^	0.81	2.19	1.21	0.01	8.54	9.28^b^
eugenol	6.76 ± 0.13	7.22 ± 0.05	6.87 ± 0.16	7.36 ± 0.20	8.11 ± 0.26	7.78 ± 0.18	21.49^b^	0.84	8.88	7.17	18.11^b^	12.26	44.38^c^
syringol	10.03 ± 1.65	42.68 ± 8.28	17.15 ± 1.25	21.95 ± 8.60	58.46 ± 14.57	26.05 ± 5.81	30.44^b^	35.60^c^	8.05	0.47	5.56	0.44	6.73
vanillin	n.d.	n.d.	n.d.	1.19 ± 0.66	2.55 ± 0.77	1.21 ± 0.53	–	–	1.94	0.00	11.76^b^	12.29	15.49^b^
4-vinylguaiacol	1.30 ± 0.01	1.43 ± 0.04	1.32 ± 0.00	1.33 ± 0.02	1.30 ± 0.00	1.33 ± 0.01	4.00	7.19^b^	5.11	0.03	8.20	11.83	7.40
**total volatile phenols**	**27.71 ± 3.33**	**79.03 ± 11.24**	**35.80 ± 1.58**	**40.89 ± 13.00**	**86.18 ± 19.40**	**43.33 ± 7.70**	39.31^c^	14.44^b^	6.28	0.08	2.89	0.06	2.75
**total volatile compounds**	**81198.93 ± 1923.57**	**96621.69 ± 9010.38**	**69037.23 ± 4755.53**	**162321.82 ± 22491.60**	**179518.28 ± 22548.24**	**236016.08 ± 6435.94**	9.05	16.86^b^	0.23	29.77^c^	38.74^c^	12.31	1306.22^d^

a C-B, control wine, at bottling time; D1-B, wine elaborated with 12 g/L of SEGs, at bottling time; D2-B, wine elaborated with 24 g/L of SEGs, at bottling time; C-6m, control wine after 6 months in bottle; D1-6m, wine elaborated with 12 g/L of SEGs, after 6 months in bottle; D2-6m, wine elaborated with 24 g/L of SEGs, after 6 months in bottle. The mean values (*n* = 4) are shown with their standard deviation. For each compound, significant differences between treated wines with its respective control at bottling time and about itself after 6 months in the bottle are indicated according to Fisher’s LSD test.

b *p*-value < 0.05.

c *p*-value < 0.01.

d *p*-value < 0.001.

**Table 2:** Low-Molecular-Weight Phenolic Compounds (mg/L) of Wines^a^

	C-B	D1-B	D2-B	C-6m	D1-6m	D2-6m	C-B/D1-B	C-B/D2-B	C-6m/D1-6m	C-6m/D2-6m	C-B/C-6m	D1-B/D1-6m	D2-B/D2-6m
**Flavanols (mg/L)**													
(+) - catechin	92.72 ± 1.01	85.76 ± 0.19	85.69 ± 0.39	106.37 ± 1.87	105.54 ± 3.53	102.73 ± 0.58	91.47^b^	84.12^b^	0.09	6.94	82.60^b^	33.22^b^	1195.73^d^
(−) - epicatechin	301.83 ± 0.61	335.66 ± 1.15	356.44 ± 3.42	560.79 ± 25.38	543.85 ± 4.62	375.42 ± 44.11	1343.27^d^	492.99^c^	0.86	26.53^b^	208.03^c^	1909.85^d^	0.37
epigallocatechin gallate	n.d.	n.d.	n.d.	44.58 ± 0.40	53.89 ± 0.32	48.60 ± 0.64	–	–	646.00^c^	55.97^b^	24267.90^d^	30939.06^d^	4.85
procyanidin B2	7.83 ± 0.36	9.61 ± 0.33	14.18 ± 3.14	14.25 ± 1.33	15.12 ± 1.55	9.12 ± 0.85	25.79^b^	8.06	0.36	21.15^b^	43.20^b^	12.40^b^	11463.84^d^
**total flavanols**	**402.39 ± 1.98**	**431.03 ± 1.01**	**456.31 ± 6.95**	**725.99 ± 25.51**	**664.50 ± 2.96**	**487.27 ± 44.49**	330.84^c^	111.11^c^	0.17	27.49^b^	319.78^c^	8434.21^d^	6.24
**Phenolic Acids (mg/L)**													
ellagic acid	39.23 ± 0.53	38.81 ± 0.73	35.23 ± 0.08	40.95 ± 0.27	42.79 ± 0.90	42.65 ± 0.18	0.44	111.68^c^	39.44^b^	13.45	16.90	36.52^b^	305.11^c^
gallic acid	18.02 ± 0.34	20.28 ± 0.11	18.62 ± 0.96	25.02 ± 0.27	26.42 ± 0.90	25.75 ± 0.18	76.90^b^	0.69	4.41	10.12	509.17^c^	47.19^b^	105.53^c^
protocatechuic acid	0.63 ± 0.03	0.76 ± 0.01	0.66 ± 0.02	1.95 ± 0.17	1.40 ± 0.05	1.32 ± 0.06	23.57^b^	1.27	20.02^b^	25.53^b^	119.98^c^	161.19^c^	190.82^c^
syringic acid	4.79 ± 0.36	5.41 ± 0.30	4.66 ± 0.08	4.45 ± 0.06	5.02 ± 0.06	4.58 ± 0.09	5.74	0.25	82.33^b^	2.63	1.64	18.24^b^	0.93
*t*-caffeic acid	0.78 ± 0.04	1.49 ± 0.07	1.07 ± 0.04	n.d.	n.d.	n.d.	144.90^c^	56.87^b^	–	–	587.55^c^	425.32^c^	450.30^c^
*t*-caftaric acid	22.37 ± 0.08	21.69 ± 0.09	20.14 ± 0.02	21.16 ± 0.48	20.99 ± 0.24	19.86 ± 0.09	57.90^b^	1354.09^d^	0.19	13.85	12.20	13.68^b^	17.98^b^
*t*-coumaric acid	n.d.	2.88 ± 0.17	1.55 ± 0.09	n.d.	n.d.	n.d.	602.63^c^	587.55^c^	–	–	–		
*t*-coutaric acid	3.16 ± 0.12	3.22 ± 0.04	3.16 ± 0.01	3.92 ± 0.05	3.42 ± 0.07	3.24 ± 0.06	0.51	0.00	60.80^b^	153.47^c^	65.54^b^	13.22	4.44
4-hydroxybenzoic acid	1.37 ± 0.01	1.84 ± 0.09	1.83 ± 0.14	0.45 ± 0.27	0.46 ± 0.19	0.34 ± 0.16	50.15^b^	22.05^b^	0.00	0.25	23.44^b^	53.31^b^	97.47^b^
vanillic acid	1.78 ± 0.16	1.85 ± 0.14	1.64 ± 0.17	0.92 ± 0.05	0.97 ± 0.14	0.61 ± 0.11	0.23	0.67	0.29	75.83^b^	51.00^b^	23.47^b^	73.86^b^
**total phenolic acids**	**92.12 ± 0.05**	**98.24 ± 0.97**	**88.55 ± 0.59**	**98.50 ± 0.21**	**101.47 ± 1.24**	**98.35 ± 0.42**	78.26^b^	70.95^b^	8.79	2.10	1926.22^d^	4.18	359.32^c^
**Stilbenes (mg/L)**													
*t*-resveratrol	0.66 ± 0.03	3.73 ± 0.52	2.11 ± 0.32	n.d.	2.41 ± 0.04	1.24 ± 0.08	68.63^b^	42.18^b^	6228.56^d^	473.80^c^	1315.77^d^	11.68	14.44
viniferine	n.d.	n.d.	n.d.	0.38 ± 0.03	1.04 ± 0.02	0.71 ± 0.00	–	–	774.80^c^	301.56^c^	408.56^c^	3173.04^d^	74024.98^d^
**total stilbenes**	**0.66 ± 0.03**	**3.73 ± 0.52**	**2.11 ± 0.32**	**0.38 ± 0.03**	**3.45 ± 0.06**	**1.95 ± 0.08**	68.63^b^	42.18^b^	3986.10^d^	746.28^c^	110.96^c^	2.39	0.50
**Anthocyanins (mg/L)**													
delphinidin 3-*O*-glucoside	21.54 ± 0.05	18.88 ± 0.15	15.66 ± 0.15	14.06 ± 0.18	13.88 ± 0.36	9.33 ± 0.13	586.03^c^	22339.64^d^	0.40	918.97^c^	3338.72^d^	300.32^c^	4376.26^d^
petunidin 3-*O*-glucoside	29.72 ± 0.19	24.07 ± 0.13	19.72 ± 0.07	17.29 ± 0.14	17.03 ± 0.03	10.70 ± 0.14	1165.64^d^	4893.23^d^	6.67	2136.34^d^	5483.18^d^	4545.47^d^	6451.41^d^
peonidin 3-*O*-glucoside	14.02 ± 0.08	11.94 ± 0.04	10.32 ± 0.01	n.d.	n.d.	n.d.	1175.67^d^	4465.62^d^	–	–	64491.02^d^	137565.62^d^	22282705.99^d^
malvidin 3-*O*-glucoside	250.24 ± 1.23	216.92 ± 1.24	184.40 ± 0.77	137.56 ± 0.13	147.18 ± 1.77	78.00 ± 0.63	691.94^c^	3858.53^d^	58.64^b^	16955.77^d^	15119.12^d^	1831.82^d^	22952.85^d^^b^
malvidin 3-(6′-acetyl)-glucoside	14.90 ± 1.05	11.84 ± 0.93	11.53 ± 0.73	64.23 ± 0.68	61.84 ± 0.44	34.14 ± 0.60	9.51	13.89	17.47^b^	2204.54^d^	3109.59^d^	2396.75^d^	1146.64^d^
malvidin 3-(6′-*t*-caffeoyl)-glucoside	120.60 ± 0.70	93.27 ± 0.50	90.65 ± 0.71	n.d.	n.d.	n.d.	721.56^c^	1806.42^d^	–	–	59733.05^d^	6592.31^d^	32474.28^d^
petunidin 3-(6′-*p*-coumaroyl)-glucoside	5.05 ± 1.25	4.79 ± 0.11	4.67 ± 0.01	n.d.	n.d.	n.d.	1.22	2.96	–	–	530.12^c^	2117.11^d^	367916.41^d^
malvidin 3-(6′-*p*-coumaroyl)-glucoside	29.05 ± 0.33	18.82 ± 0.16	17.18 ± 0.20	14.82 ± 0.26	11.76 ± 0.08	7.38 ± 0.04	1569.66^d^	379.00^c^	255.94^c^	1599.90^d^	2323.48^d^	2397.27^d^	300.88^c^
**total anthocyanins**	**493.11 ± 0.51**	**408.53 ± 4.02**	**362.13 ± 0.30**	**247.98 ± 1.13**	**251.69 ± 1.73**	**139.55 ± 0.91**	869.21^c^	3479.07^d^	6.46	11174.05^d^	78158.31^d^	2078.04^d^	9500.51^d^
**Flavonols (mg/L)**													
myricetin 3-*O*-galactoside	0.97 ± 0.01	0.85 ± 0.03	0.84 ± 0.01	n.d.	n.d.	n.d.	32.89^b^	6305.34^d^	–	–	395262.35^d^	981.49^d^	3747838.86^d^
myricetin 3-*O*-glucuronide	2.83 ± 0.06	1.50 ± 0.01	1.48 ± 0.03	2.35 ± 0.01	1.55 ± 0.07	1.59 ± 0.02	1091.19^d^	825.30^c^	4246.61^d^	1983.04^d^	137.45^c^	177.36^c^	15.47
quercetin 3-*O*-glucuronide/glucoside	4.86 ± 0.07	3.59 ± 0.06	3.52 ± 0.02	3.61 ± 0.11	2.72 ± 0.08	2.63 ± 0.02	372.49^c^	610.99^c^	93.45^b^	160.56^c^	183.19^c^	104.61^c^	1733.05^d^
laricitrin 3-*O*-glucuronide/galactoside	1.37 ± 0.01	1.03 ± 0.02	1.14 ± 0.01	n.d.	n.d.	n.d.	359.81^c^	746.52^c^	–	–	33028.81^d^	2438.89^d^	124363.18^d^
syringetin 3-*O*-glucoside	1.91 ± 0.01	1.85 ± 0.03	1.55 ± 0.03	1.89 ± 0.22	1.83 ± 0.05	1.63 ± 0.01	8.84	261.16^c^	14.68	243.10	2.23	2.62	11.74
myricetin 3-*O*-glucoside	1.73 ± 0.08	1.21 ± 0.05	0.94 ± 0.03	1.26 ± 0.02	1.12 ± 0.04	0.98 ± 0.04	63.45^b^	175.48^c^	21.24^b^	82.59	63.24^b^	4.82	1.72
quercetin 3-*O*-glucoside	2.89 ± 0.18	1.52 ± 0.11	1.02 ± 0.04	1.86 ± 0.02	1.38 ± 0.02	1.16 ± 0.03	79.97^b^	206.39^c^	844.25^c^	976.30^c^	64.41^b^	1.58	20.61^b^
**total flavonols**	**16.57 ± 0.38**	**11.55 ± 0.14**	**10.50 ± 0.15**	**10.96 ± 0.06**	**8.59 ± 0.01**	**8.00 ± 0.07**	305.68^c^	440.11^c^	2748.00^d^	2087.05^d^	419.53^c^	570.41^c^	485.66^c^
**total phenolic compounds**	**1004.84 ± 2.95**	**953.08 ± 6.68**	**919.61 ± 1.12**	**1084.14 ± 24.08**	**1083.60 ± 3.38**	**783.71 ± 43.15**	100.61^c^	109.87^c^	0.00	73.93^b^	21.36^b^	529.11^c^	18.61^b^

a C-B, control wine, at bottling time; D1-B: wine elaborated with 12 g/L of SEGs, at bottling time; D2-B, wine elaborated with 24 g/L of SEGs, at bottling time; C-6m, control wine after 6 months in bottle; D1–6m, wine ela6morated with 12 g/L of SEGs, after 6 months in bottle; D2-6m, wine elaborated with 24 g/L of SEGs, after 6 months in bottle. The mean values (*n* = 4) are shown with their standard deviation. For each compound, significant differences between treated wines with its respective control at bottling time and about itself after 6 months in the bottle are indicated, according to Fisher’s LSD test.

b *p*-value < 0.05.

c *p*-value < 0.01.

d *p*-value < 0.001.

**Figure 1:**
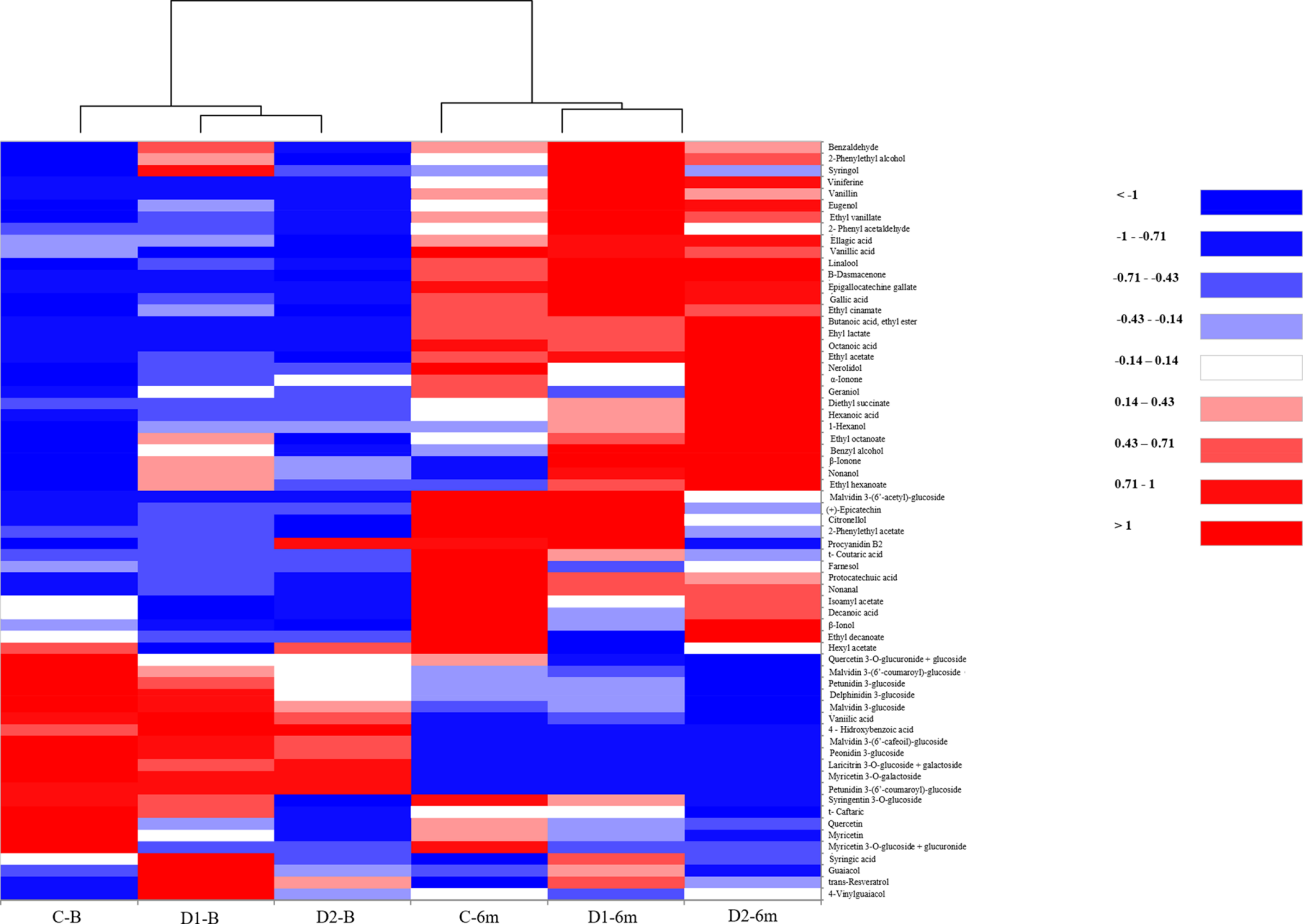
Heatmap and dendrogram based on phenolic and volatile composition of wines. Relative concentrations of each compound are represented via a chromatic scale with the darkest red corresponding to the highest concentrations and the darknest blue corresponding to the lowest concentrations.

For a rapid visual assessment of the similarities and differences between wines, a heatmap with dendrogram representation was performed (Figure 1), corresponding to the graphical representation of the data of wines from Tables 1 and 2 and the volatile and phenolic compositions, respectively. The chromatic scale of the heatmap refers to the relative amount of each volatile or phenolic compound (from dark blue, minimum, to dark red, maximum), whereas the dendrogram reveals clustering between the wines under study, gathering them according to their volatile and phenolic profile similarities. According to this, it was possible to observe two main clusters corresponding to the time in bottle (bottling and six months of bottle aging) and, considering a lower level of grouping, another four clusters corresponding to the control and SEG wines at each analysis moment.

The heatmap shows the clear evolution in the chemical composition of wines during bottle aging, going from wines with a higher content of low-molecular-weight phenolic compounds at bottling (blue color) to having a higher content of aromatic compounds after six months of bottling (red color).

In terms of volatile compounds, considering their grouping by chemical families, it was observed that their concentration increases or remains constant with bottling, depending on the wine, but none of them decreased with bottle aging, highlighting the increase of the ester family, which agrees with other works about the evolution of Cabernet Sauvignon wines after bottling.^19^ In contrast, in the case of phenolic compounds, a decline in anthocyanins, stilbenes, and flavonols was found, but a significant increase of flavanols and phenolic acids was observed in most wines.

Esters were the most abundant group of volatile compounds in all wines, which are primarily responsible for aroma,^20^ especially ethyl esters of fatty acids and acetates with a higher alcohol content. At bottling, the highest content of this family of compounds was observed in the control wine, followed by D1-B wines. Bottle aging contributed positively to the total content of these compounds, which increased in all wines, but more significantly when the highest dose of SEGs was used (Table 1). The formation of ethyl esters of organic acids is one of the principal chemical changes that occur during wine aging and these esterification reactions favor the development of aroma and taste in wine.^11,21^ As such, the increase of esters in wine during bottle aging has been widely demonstrated.^21^ In this work, wines in contact with the highest doses of SEGs (D2-6m) showed a more significant increase in ethyl esters, mainly ethyl lactate and diethyl succinate, which could suggest that a high dose of SEGs enhances the acid esterification. Moreover, other compounds that also increase their concentration with bottle maturation, such as ethyl butyrate, ethyl hexanoate, or ethyl octanoate, are associated with flavor attributes such as fruity, floral, and sweet notes,^22^ which could have a potential sensory impact on the wines. Moreover, the content of isoamyl acetate (pineapple aroma^23^) increased in all wines but more significantly in the D2-6m wine. Different studies associate the increase in acetate esters with acid-catalyzed reactions of fatty acid esters during bottle aging, and as a result, some acetate esters were produced.^24,25^

Alcohols were the second more abundant group of compounds and were dispersed throughout the heatmap, showing a change from dark blue to dark red in wines after bottle aging (Figure 1). At bottling, D1-B wine showed the highest total content of these compounds, followed by the control and D2-B wines. However, after bottle aging, both wines elaborated in contact with SEGs showed the highest alcohol content, mainly D1-6m wine (Table 1), behavior that agrees with previous research.^15^ The higher concentrations of alcohols found in SEG wines could be associated with the amino acid composition of vine-shoots,^26^ which could increase the concentration of amino acids in the musts and enhance the synthesis pathways of alcohols. The most abundant alcohols were 2-phenylethanol and 1-hexanol (Table 1), which are characterized by a floral and rose odor in the first case and grass notes in the second. However, only 2-phenylethanol slightly exceeded its perception threshold (10 000 μg/L) in D1-6m wine.

Regarding acids, even though chromatic differences were observed in the heatmap, the statistical treatment did not show significant differences in the total content of wines elaborated in contact with SEGs, in contrast with control, at any of the analysis times (Table 1). However, in terms of individual compounds, decanoic acid was lower in SEGs wines at bottling time, and hexanoic acid was three times higher in D2-6m wines compared with the control. The increase of this acid in wines elaborated with 24 g/L added after malolactic fermentation has already been described by Cebrián-Tarancón et al.^1^

Norisoprenoids were positively affected by bottle aging, with an increase in the total content of these compounds in all wines and reaching levels of 34.07 μg/L in the D2-6m, which is higher than in control wine (Table 1). This group was mainly represented by β-ionol, which did not show differences after bottle aging between control and SEG wines. This compound is a precursor of β-ionone, a norisoprenoid associated with a violet floral, berry, and balsamic aroma,^1^ which was found in higher concentrations in wines elaborated in contact with SEGs, according to previous works associated with the use of this enological additive^1^ and whose increase could be associated with bottle aging. β-Damascenone, a compound with a baked apple and dry plum aroma,^23^ did not show significant differences among wines studied after bottling but may directly contribute to the sensory profile of wines due to synergistic effects with other compounds, since it presented concentrations between 6.14 μg/L and 9.00 μg/L (Table 1), which are higher than its perception odor threshold (0.05 μg/L).

Volatile phenols are a group related to the “*wood nature*” of vine-shoots, since they are a characteristic product of lignin thermal decomposition.^16^ Therefore, the contribution of vine-shoots to this group of compounds was to be expected, being more significant when the lowest dose of SEGs used was nearly three times higher than that of the control (Table 1). These higher contents were mainly due to the syringol contribution, which involves a compound characterized by smoke notes that reached levels of 42.68 and 58.46 μg/L in D1 wines at the first and second moment of analysis, respectively. Guaiacol, which is also associated with smoky and toasted notes,^27^ showed important concentrations in D1-B, significantly higher than those in the control wine. However, these differences disappeared with bottle aging and all wines showed similar concentrations. It is important to note the behavior of vanillin, which was not found at the beginning of the analysis but was detected after bottle aging, although it did not show significant differences between wines (Table 1).

Terpenes have been described as one the most important compounds in wine, which contribute to the varietal characteristics of wine, thanks to their flowery and sweet aroma nature.^28^ In this work, the total content of terpenes was lower at the first moment of analysis, with no difference found between wines (Table 1). However, bottle aging increased their content in all the wines, although only significantly so in D2-6m wine, with respect to the control wine. In this way, the higher content in wines according to the aging time could be attributed to the glycosidic bonds of precursor compounds being broken during the bottling period and their free form being released into the wine, thus increasing its final concentration and improving the varietal aroma character of the wines.^13^ Geraniol was the most abundant compound of this group, characterized by notes of geranium and rose^29^ and with significantly higher levels in D1-B than in control wine. Linalool and nerolidol, which are compounds associated with jasmine, orange blossom, and sweet or floral aromas^23,30^ and located in the middle of the heatmap, were higher in both SEG wines at bottling time, while these differences disappeared with permanence in the bottle (Table 1).

Regarding low molecular weight, phenolic compounds were also analyzed (Table 2). Anthocyanins, mainly those located in the lower half of the heatmap (Figure 1), presented the highest decrease in the total content (61.46%) when wines were in contact with the highest dose of SEGs (24 g/L). Malvidin-3-*O*-glucoside, the monomeric anthocyanin, was the most affected by bottle aging, decreasing to 57.70%. However, when a dose of 12 g/L of SEGs was used, the decrease of these compounds was lower than that in the control wine behavior previously reported by Cebrián-Tarancón et al.^1^ Moreover, it is important to note the degradation of peonidin-3-*O*-glucoside, malvidin 3-(6-*t*-caffeoyl)-glucoside and petunidin 3-(6′-*p*-coumaroyl)-glucoside, which was undetected after bottle aging, as well as the significant increase of malvidin 3-(6′-acetyl)-glucoside, mainly in D1-B wine (Table 2). Although bottle aging of wines elaborated in contact with SEGs has not been previously studied, the decrease in the anthocyanin content of Cabernet Sauvignon wines during storage has already been observed by other authors,^31^ who suggested that this decrease is consistent with the involvement of these compounds in numerous condensation reactions during the storage period as well as in hydrolytic reactions. Moreover, the fact that the evolution of wines with an SEG concentration of 12 g/L was better than that of the control could be attributed to the fact that this lower dose influences the vine-shoots/wine balance, facilitating the formation of more stable structures, while higher doses of SEGs would break the balance, so that the wood sorption could favor the reduction of the content of these compounds, as recently observed Cebrián-Tarancón et al.^1^ when studying the chemical exchange in the vine-shoot/wine system.

Flavanols, dispersed along the heatmap, had a positive behavior with bottle time, increasing its concentration in control wine and when an SEG concentration of 12 g/L was used (D1-6m) but remained constant in the case of higher doses of SEGs (D2-6m). As previously described in other works,^1^ the most important flavanol was (−)-epicatechin, located in the middle of the heatmap (Figure 1), with levels close to 375.42 mg/L in D2-6m wine to 560.79 mg/L in C-6m. This behavior contrasts with the results from other authors, who found that flavanols decline during aging.^32−34^ These results could be due to the interflavanic bond cleavage of proanthocyanidins during aging, generating smaller-size polymers and thus increasing the concentration of available end units, as suggested in Monagas et al.^35^ However, in D2-6m wines, this decrease could be associated with a sorption by SEGs due to a greater SEGs/wine contact surface or even that a higher dose improves anthocyanin-flavanol reactions. As for the rest of the compounds, procyanidin B2 showed a similar trend to (+)-epicatechin, while (+)-catechin increased its concentration in all the wines, with no differences found between the control and treated wines. Epigallocatechin gallate only was detected after bottle aging, which suggests the de novo formation of compounds during bottle aging.

Phenolic acids were the third most abundant group of compounds, with the main ones being ellagic and gallic acids (Table 2). Their increase in wines elaborated in contact with SEGs could be associated with a release from wood since these are the most abundant phenolic acids in toasted vine-shoots, as was already reported.^36^ Hydroxycinnamic, *trans*-caffeic, and *trans*-coumaric acids showed the highest concentrations in wines elaborated in contact with SEGs at bottling time. This was to be expected since these compounds are part of the lignin structure,^37^ and the chemical composition of vine-shoots is characterized by an important lignin fraction whose content is ∼38.5%.^38^ However, a significant decrease in bottle time was revealed for these in all wines. Accordingly, their corresponding ester, *trans*-caftaric and *trans*-coutaric acids were detected in both analysis moments. The first analysis moment experienced a slightly decreased concentration in wines elaborated with SEGs, which could be associated with a sorption from toasted vine-shots,^39^ while the second one remained constant in treated wines but increased in control wines, possibly due to esterification reactions from *trans*-coumaric acid.

Flavonols, another family of compounds with a key role in the stabilization of the red wine color,^40^ showed lower concentrations in wines elaborated in contact with SEGs, in agreement with Cebrián-Tarancón et al.^1^ and negatively affected by bottle aging (Table 2). In detail, these compounds exhibited quite different behaviors. Quercetin 3-*O*-glucuronide/glucoside were the most abundant flavonols, although their content decreased in all wines after bottle aging. Myricetin 3-*O*-galactoside and laricitrin 3-*O*-glucuronide/galactoside were undetectable after six months in bottle, while syringentin 3-*O*-glucoside remained constant in all wines with aging. Finally, the behavior of myricetin 3-*O*-glucuronide, myricetin 3-*O*-glucoside and quercetin 3-*O*-glucoside were dependent on the type of wine, although, in all cases, their levels in the control wine decreased. As stated above, the decrease in the total content of flavonols could be due to reactions with other compounds for the formation of more stable polymeric pigments or, in some cases, to wood sorption, as recently reported by Cebrián-Tarancón et al.^1^

Vine-shoots can be considered the actual raw material for *trans*-resveratrol isolation,^41−44^ so the significant contribution of SEGs to wines related to this compound was expected and has been previously reported in research where vine-shoots have been used as enological additives.^1−3^ In this work, the *trans-*resveratrol content in D1-B wine was 3.73 mg/L at bottling time and three times higher in D2-B wines (2.11 mg/L) than in the control wine. However, although bottle aging decreased the concentration of this compound to undetected levels in the control wine, its concentration in SEG wines remained higher than normally detected in red wine.^45,46^ It is known that *trans*-resveratrol has numerous potential biological activities,^47^ so the results again support the potential benefits of these wines.

### 3.2 Effect of SEGs in the Sensory Profile of Wines

**Figure 2:**
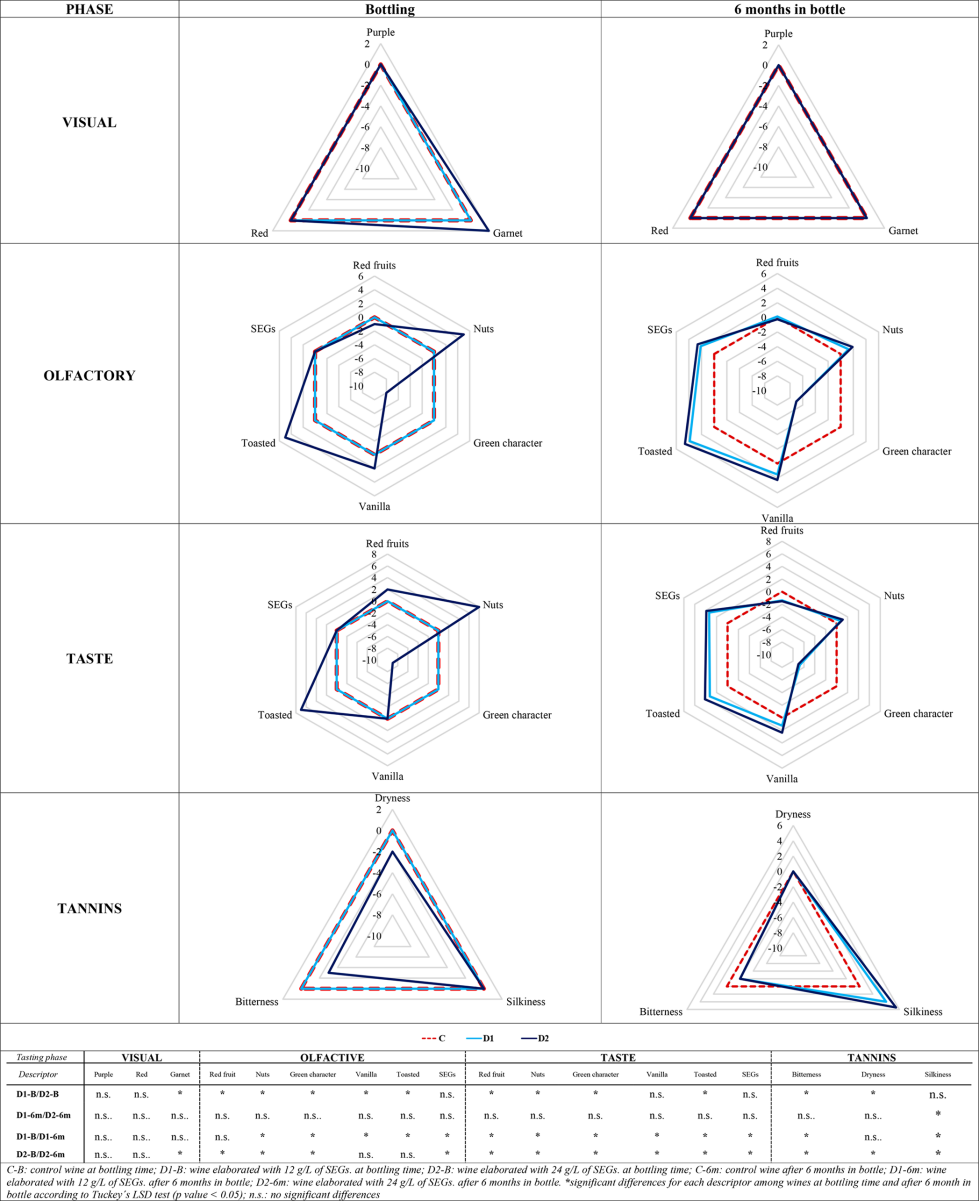
Sensory profile of wines made in contact with SEGs at bottling and after 6 months in bottle. Differences with respect to the control wine are shown.

In order to explore the effect of the addition of SEGs on the sensory profile of wines, the evaluation of different Cabernet Sauvignon wines was described by the panelist at bottling time and after six months of bottle aging. Visual, olfactory, and taste phases were evaluated, and a total of 12 descriptors were discussed, showing the results as differences with respect to the control wine in spider charts (Figure 2). The one-way analysis of variance (ANOVA) shows the significant differences between the wines with respect to the control wines obtained with different doses at the same analysis time (D1-B/D2-B and D1-6m/D2-6m) and the same dose at different analysis times (D1-B/D1-6m and D2-B/D2-6m). Positive values indicate a higher perception with respect to control, while negative values indicate a lower perception, with respect to control. In order to help understand the spider charts (Figure 1), the zero line has been indicated (with a dashed line) to show the location of the normalized control wine.

Regarding the visual phase, at bottling time, wines from both SEG doses showed a similar score for violet and red tones to that of the control wine, while slight differences in garnet tones were observed in D2-B. However, in contrast to other woods used in the enology,^48^ bottle aging smoothed out these differences, and all wines showed a similar profile in the visual phase.

Similar descriptors were evaluated in the olfactory and taste phases (red fruits, SEGs, nuts, toasted, vanilla, and green character), and the profiles obtained in both were very similar. In the tasting phase carried out at bottling time, only differences in the taste of wines elaborated in contact with 24 g/L of SEGs were found with respect to the control wine, except in the case of SEG attributes. However, with bottle time, differences with respect to the control wine were also detected when the lower dosage of SEGs was used (D1-6m wine). At this time, the descriptors that showed higher scores than the control wine were “*toasted*”, “*vanilla*,” and “*nuts*”. In the olfactory phase, toasted descriptor was 5-fold greater than the control wine at both tasting times, but it had a greater intensity at bottling time during the tasting phase. However, bottle aging enhanced the differences in the wines elaborated with 12 g/L SEGs, with respect to the control, equalizing the intensities in both wines with treatment. The increase of the toasted aroma in wines because of winemaking with SEGs had already been suggested in other works, based on the volatile compounds of these wines,^1^ and this agrees with the sensory profile defined by tasters of Malbec wines elaborated in contact with vine-shoot chips.^4^

The “*nuts*” attribute detected by panelists at tasting time was associated with hazelnut and almond shells and showed similar behavior to the toasted descriptor, with the highest intensity in wines elaborated in contact with 24 g/L of SEGs at the beginning of tastings—five and eight times more intense than the control in the olfactory and taste phases, respectively. However, after bottle aging, the intensity was softened in D2-6m wines and, by contrast, was slightly detected in D1-6m wines by the tasters. The presence of this aroma in wine has been previously described in wines aged in *Q. petraea* barrels^49^ and is associated with certain aldehydes, such as benzaldehyde.^50^

As for the “*vanilla*” descriptor, wines elaborated in contact with the highest dose of SEGs (24 g/L) had a higher score than the control during the first tasting in the olfactory phase. However, this descriptor was not perceived in D1 wines until six months after bottling. By contrast, in the tasting phase, bottle aging slightly enhanced these notes in treated wines and was more intense in the D2 wine.

Regarding the “SEGs” attribute, aromatic notes associated with the contribution of SEGs to the wines did not show significant differences with respect to the control wine in the first tasting in any of the tasting phases, but bottle aging encouraged the intensity of this descriptor in wines elaborated in contact with SEGs in a similar way for both doses. This attribute had already been described by Cebrián-Tarancón et al.,^1^ who, taking into account the chemical profile of wines, associated this with a “*sweet woody*” that included descriptors such as spiced (eugenol), sweet, and balsamic (ethyl cinnamate) or toasted (guaiacol) and vanilla notes (vanillin) in its odorant series, the latter being described above.

It is important to note the significant decrease in the “*green character*” attribute in wines elaborated in contact with SEGs, mainly in the wines treated with the highest dose, where this reduction was appreciable from the first tasting, both during the olfactory and taste phases. The “*green character*” is a multivariate character negatively correlated with wine preferences and associated with both aroma and mouthfeel sensations, such as vegetal, astringency, green or dry tannins^51^ and which they associate with interactions between isoamyl alcohol, anthocyanin-derivate fraction, and/or tannins. The fact that toasted vine-shoots modify the anthocyanin profile of wines along the contact (Table 2) could reduce these interactions and consequently lead to a lower perception of the green character by tasters.

Finally, Table S4 in the Supporting Information shows the opposite behavior of the SEGs and *green character* descriptors in agreement with the patent PCT/EP2021/082717.^52^

Finally, the “*red fruit*” descriptor showed the greatest differences between tasting phases. At bottling time, tasters only detected differences with respect to the control in D2 wine, with less intensity than the control in the olfactory phase and highest intensity during the tasting phase. However, after bottle aging, treated wines did not show differences from the control wines in the olfactory phase but were below the control in the tasting phase. Pineau et al.^53^ suggested that the type of berry fruit is related to the specific profile of ethyl esters in wines, so this lower perception at bottling time could be due to a lower concentration of certain esters such as ethyl butyrate or ethyl lactate, which are both associated with strawberry or raspberry fruit notes.

Regarding tannins, at bottling time, differences with respect to the control only were found in wines elaborated with the highest dose of SEGs, 24 g/L, which were less bitter and less dry than the control wine. However, after bottle aging, all wines were similar in relation to dry mouthfeel sensation, but those elaborated in contact with SEGs were less bitter and silkier than the control. No differences were found between doses in the first sensation, while the second was slightly higher when 24 g/L of SEGs were used. The previously mentioned anthocyanin-flavanol reactions not only modified the wine color but also brought about changes in other attributes, such as astringency and bitter flavors, which decrease and, thereby, round out or soften the wine.^33^ The silky sensation has been recently negatively correlated with some monomeric anthocyanins such as delphinidin 3-*O-*glucoside, petunidin 3-*O*-glucoside, or malvidin 3-*O*-glucoside.^54^ This suggests that the degradation of these anthocyanins, which decreases their concentrations both for the use of SEGs and for the subsequent bottle aging, could be one of the factors for increasing wine silkiness during aging.

**Figure 3:**
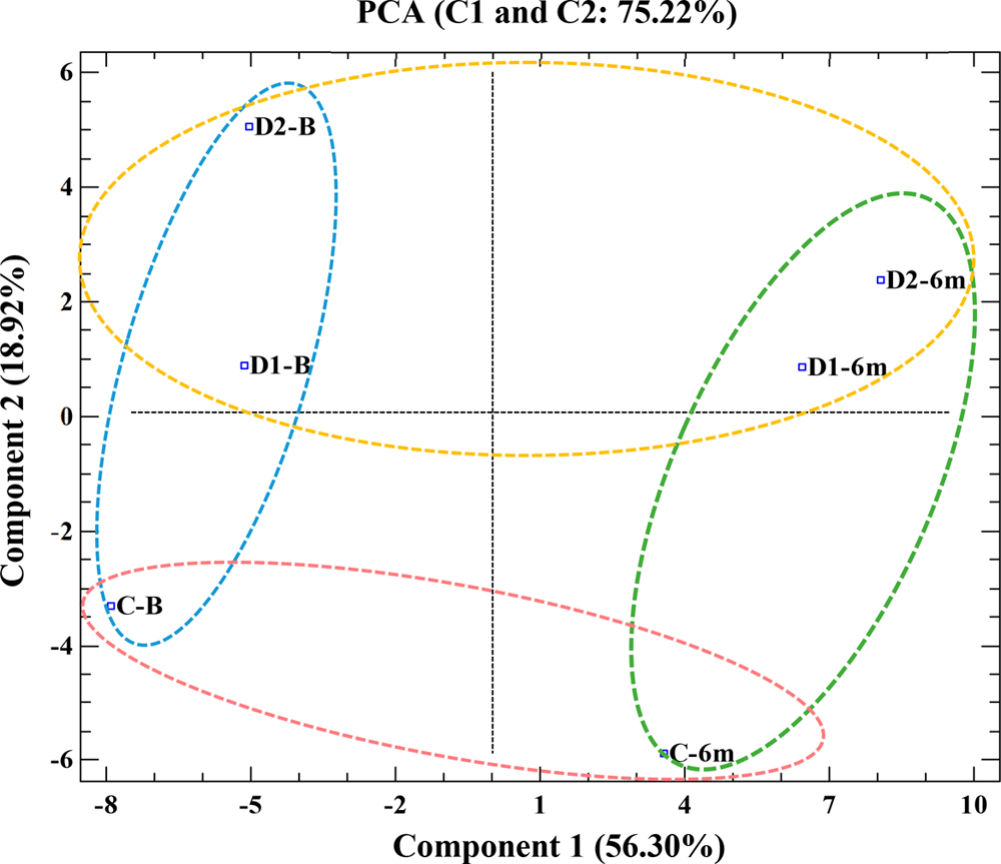
Principal component analysis (PCA) based on phenolic and volatile composition, and sensory profile of wines.

Finally, in an attempt to determine the relationships between the chemical and sensory differences observed in the wines, as well as the variables with the greatest influence in this differentiation, all of the wines were positioned in a principal component analysis (PCA) based on the volatile and phenolic composition and sensory profile of wines (Figure 3). Two component functions were constructed, which explained 75.22% of the total variance, with 56.30% in variance found for Component 1, which separated the samples according to the moment of bottling time, and 18.92% in variance found for Component 2, which separated wines in contact with SEGs from the control. Moreover, the variable weight table for each component (Figure 3) shows that the chemical composition of wines has a higher weight in the separation within Component 1, while the sensory analysis has a higher weight in Component 2. This clustering reflects the important effect of SEGs, which agrees with the results of Cebrián-Tarancón et al.,^1^ who observed a significant cluster of SEGs wines from the control, with these differences increasing according to contact time and SEGs dose. However, the effect of bottle aging on the chemical profile of the wines is also observed in this case. Therefore, a significant modulation of the sensorial profile of wine is expected. A negative correlation was observed between the silkiness tannins of wines and some monomeric anthocyanins and between the SEGs descriptors and the green character of the wines, as previously suggested. By contrast, positive correlations were observed between the SEG descriptors and some esters, alcohols, acids, or norisoprenoids and also between red fruit attributes and some esters (see Figure S1 in the Supporting Information). However, it is difficult to find a direct relationship between the aromatic and phenolic composition of wines and their sensory profiles since numerous factors are appointed as determinants of wine evolution and organoleptic quality during the post-bottling process. In particular, various components in red wines could affect volatile release and aroma perception definition, as already reported by several researchers.^55−57^ Furthermore, this is the first study of the sensory impact of Cabernet Sauvignon SEGs in wines of the same variety when they are used as an enological tool, so the possible synergies or antagonisms between compounds and the effect that the nonvolatile matrix could have are still unknown, being required more future studies on this topic.

In conclusion, the results of this work show that the use of pruned toasted vine-shoots in Cabernet Sauvignon wines as a new enological tool (SEGs) produces wines with a differentiated character. Regarding wines chemical composition, aging for six months in the bottle results in a decrease in low-molecular-weight phenolic compounds, mainly anthocyanins and flavonols, an increase in some volatile compounds, mainly esters, and the preservation of a significant amount of *trans*-resveratrol. In terms of the sensory profile, bottle aging gives rise to rounder wines with more integrated aromas but higher notes of nuts, toasted, and vanillin, as well as less bitter and silkier tannins, compared to the control wine.

## Supporting Information Available

Enological parameters of grapes at the harvest day (Table S1); enological parameters of wines at bottling time and after 6 months of bottle (Table S2); weights of the total variables (volatile and phenolic compounds, and sensory descriptors) in the first two principal components (Table S3); correlations between sensory descriptors of the olfactory and gustatory phases and volatile compounds identified in all wines (Table S4); principal component analysis (PCA): components weight (Figure S1) (PDF)
