# Supplementary material for: Evolution in the Bottling of Cabernet Sauvignon Wines Macerated with Their Own Toasted Vine-Shoots
Source: J Agric Food Chem. 2023 Mar 29;72(4):1864–77. doi: 10.1021/acs.jafc.2c08978 (PMC10835714; doi:10.1021/acs.jafc.2c08978)

SUPPORTING INFORMATION

Table S1. Enological parameters of grapes at the harvest day.

ºBrix	23.6 ± 0.06
Probable alcohol degree	13.9 ± 0.05
Total acidity (g/L of tartaric acid)	5.80 ± 0.21
pH	3.58 ± 0.11

Table S2. Enological parameters of wines at bottling time and after 6 months of bottle.

	Bottling	6 months
Alcoholic degree (%, v/v)	13.92 ± 0.42 a	14.51 ± 0.30 a
Total acidity (g/L of tartaric acid)	4.29 ± 0.24 a	4.60 ± 0.26 a
pH	3.83 ± 0.12 a	3.87 ± 0.11 a
Colour intensity	11.26 ± 0.57 b	9.85 ± 0.46 a

For each parameter, significant differences before and after SEGs contact are examined using one-way ANOVA and are indicated according to Tuckey´s LSD test (p value < 0.05). The mean values (n = 6) are shown with their standard deviation

Table S3. Weights of the total variables (volatile and phenolic compounds, and sensory descriptors) in the first two principal components.

	**Component 1 (56.30%)**	**Component (18.92%)**
Toasted_T	0.0280	0.2110
Toasted_O	0.0638	0.2029
Nuts_O	-0.0043	0.2013
Vanilla_O	0.0706	0.1985
Nuts_T	-0.0358	0.1773
t-Resveratrol	-0.0301	0.1604
β-Ionone	0.1019	0.1449
Nonanol	0.1040	0.1444
Garnet	-0.1027	0.1384
Ethyl Hexanoate	0.1088	0.1109
1-Hexanol	0.1224	0.0983
4 - Hidroxybenzoic acid	-0.1331	0.0874
Vanilla_T	0.1164	0.0858
Silkiness_T	0.1202	0.0845
SEGs_O	0.1207	0.0834
SEGs_T	0.1208	0.0824
Benzyl alcohol	0.1250	0.0692
α-Ionone	0.1196	0.0689
Purple	-0.1412	0.0622
Red	-0.1413	0.0618
Syringic acid	-0.0479	0.0568
Hexanoic acid	0.1254	0.0552
Petunidin 3-6-cou-glu	-0.1421	0.0539
Syringol	0.0668	0.0530
Myricetin-3galac	-0.1427	0.0473
Ethyl octanoate	0.1195	0.0473
Diethyl succinate	0.1236	0.0448
Laricitrin 3-glu+galact	-0.1427	0.0424
Guaiacol	-0.0447	0.0340
Malvidin 3-6-cafe-glu	-0.1429	0.0322
Peonidin-3glu	-0.1426	0.0313
4-Vinylguaiacol	-0.0304	0.0269
Eugenol	0.1324	0.0250
Vanillic acid	-0.1416	0.0240
ProcyanidinB2	0.0527	0.0212
2-Phenylethyl alcohol	0.1134	0.0137
Benzaldehyde	0.1019	0.0131
Geraniol	0.1045	0.0127
Viniferin	0.1338	0.0100
Ethyl vanillate	0.1327	-0.0012
Linalool	0.1460	-0.0048
Ethyl butyrate	0.1415	-0.0182
Vanillin	0.1264	-0.0243
β-Dasmascenone	0.1442	-0.0276
Red fruits_T	0.0846	-0.0279
Ethyl lactate	0.1385	-0.0284
Benzene acetaldehyde	0.1036	-0.0301
Nerolidol	0.1270	-0.0332
Gallic acid	0.1428	-0.0353
Ethyl acetate	0.1381	-0.0385
Malvidin-3glu	-0.1361	-0.0412
Octanoic acid	0.1412	-0.0467
Epigallocatechin gallate	0.1423	-0.0473
Ethyl cinnamate	0.1368	-0.0508
Dryness_T	-0.1081	-0.0596

Delphinidin-3glu	-0.1320	-0.0711
Petunidin-3glu	-0.1312	-0.0755
Ellagic acid	0.1188	-0.0877
Malvidin-6-cou-glu	-0.1305	-0.0897
(-)-Epicatechin	0.1010	-0.0956
Hexyl acetate	-0.0147	-0.1050
Citronellol	0.1105	-0.1152
(+)-Catechin	0.1257	-0.1153
Malvidin 3-6-ace-glu	0.1155	-0.1157
Nonanal	0.1184	-0.1195
Quercetin 3-gluc+glu	-0.1222	-0.1226
Ethyl decanoate	0.0575	-0.1239
2-Phenylethyl acetate	0.1036	-0.1251
Protocatechuic Acid	0.1166	-0.1259
β-Ionol	0.1058	-0.1334
Red fruits_O	0.0949	-0.1511
Isoamyl acetate	0.0896	-0.1582
Bitterness_T	-0.0910	-0.1611
t-Caftaric	-0.0856	-0.1617
Decanoic acid	0.0785	-0.1618
t-Coutaric acid	0.0691	-0.1743
Green character_T	-0.0849	-0.1747
Myricetin	-0.0749	-0.1791
Green character_O	-0.0806	-0.1831
Quercetin	-0.0690	-0.1883
Farnesol	0.0473	-0.1949
Myricetin-3-glu+3-glucu	-0.0485	-0.2057
Syringetin-3-gluc	-0.0222	-0.2135

Table S4. Correlations between sensory descriptors of the olfactory and taste phases and volatile compounds identified in all wines.

	TASTE		OLFACTORY	
	**Green character**	**SEGs**	**Green character**	**SEGs**
Acids				
Hexanoic acid	-0.5420***	0.829***	-0.5336***	0.8194***
Octanoic Acid	-0.3963**	0.6691***	-0.3647**	0.6450***
Decanoic acid	0.0305	0.1341	0.0878	0.1279
Alcohols				
2-Phenylethyl alcohol	-0.2760*	0.6948***	-0.2866*	0.6527***
1-Hexanol	-0.6346***	0.8435***	-0.6321***	0.8261***
Benzyl Alcohol	-0.5172***	0.8823***	-0.5274***	0.8515***
Nonanol	-0.6222***	0.8198***	-0.6423***	0.7855***
Aldehydes				
Benzaldehyde	-0.1879	0.4597***	-0.1901	0.4012**
Benzene acetaldehyde	-0.3070*	0.5862***	-0.3164*	0.5085***
Nonanal	-0.1500	0.3387*	-0.0951	0.3214*
Esters				
Ethyl esters				
Ethyl lactate	-0.4561***	0.7831***	-0.4262**	0.7657***
Ethyl octanoate	-0.3277*	0.6856***	-0.3265*	0.6716***
Ethyl butyrate	-0.4799***	0.8234***	-0.4566***	0.7991***
Ethyl decanoate	0.1087	0.0611	0.1588	0.0793
Diethyl succinate	-0.5555***	0.8525***	-0.5443***	0.8516***
Ethyl vanillate	-0.4951***	0.7853***	-0.4894***	0.7387***
Ethyl hexanoate	-0.4828***	0.7743***	-0.4968***	0.7471***
Ethyl cinnamate	-0.3259*	0.6696***	-0.3058*	0.6341***
Acetates				
Ethyl acetate	-0.3595**	0.8059***	-0.3412*	0.7800***
Isoamyl acetate	-0.0487	0.2828	0.0044	0.2552
2-Phenylethyl acetate	-0.0675	0.3792	-0.0470	0.3320*
Hexyl acetate	0.0864	-0.3565	0.1378	-0.3499**
Norisoprenoids				
β-Ionol	-0.0786	0.3829	-0.0297	0.3753**
β-Damascenone	-0.4686***	0.8014***	-0.4467***	0.7724***
α-Ionone	-0.5514***	0.5122***	-0.5191***	0.5249***
β-Ionone	-0.6430***	0.8547***	-0.6659***	0.8313***
Terpenes				
Geraniol	-0.2093	0.3700**	-0.1808	0.3957**
Citronellol	-0.1248	0.3858**	-0.0971	0.3335*
Farnesol	0.2732	-0.1650	0.3343*	-0.1709
Linalool	-0.5238***	0.7809***	-0.5021	0.7450***
Nerolidol	-0.3559**	0.4804***	-0.3077	0.4715***
Volatile phenols				
Guaiacol	0.3836**	-0.2230	0.3466*	-0.2362
Eugenol	-0.4935***	0.8162***	-0.4939***	0.7771***
Syringol	-0.2192	0.4709***	-0.2464	0.4119**
Vanillin	-0.4663***	0.7425***	-0.4589***	0.6485***
4-Vinylguaiacol	0.4144**	-0.2445	0.4027**	-0.2302

Figure S1. Principal component analysis (PCA): weight of components.

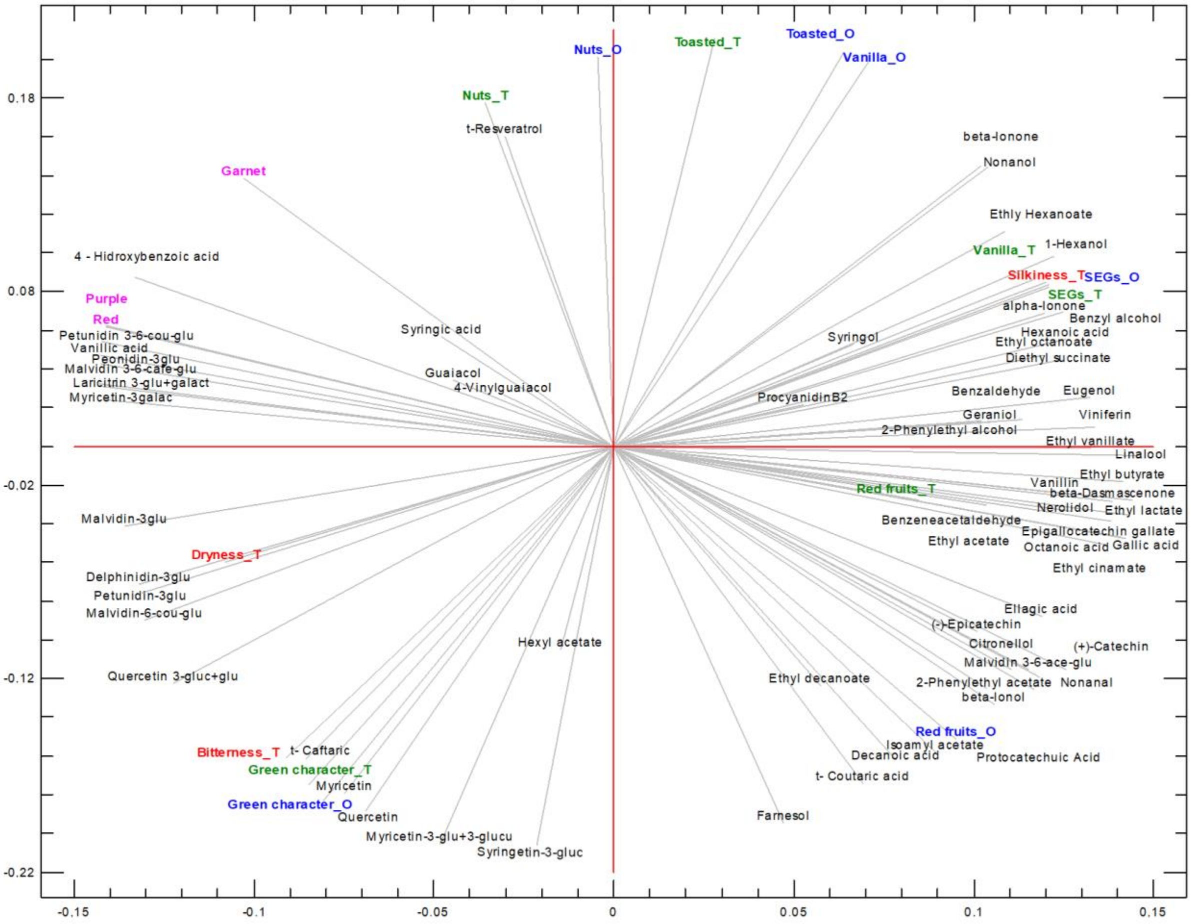

Supplement: Supplementary file 1 — jf2c08978_si_001.pdf [file jf2c08978_si_001.pdf]
